# A Dyadic Nosology for Osteogenesis Imperfecta and Bone Fragility Syndromes 2024

**DOI:** 10.1007/s00223-024-01248-7

**Published:** 2024-06-28

**Authors:** David Owen Sillence

**Affiliations:** 1 Specialities of Genomic Medicine and Paediatrics and Adolescent Health, Children’s Hospital Westmead, Sydney University Clinical School, Westmead, NSW 2145 Australia; 2 Department of Genetic Medicine, Honorary Emeritus Consultant, Westmead Hospital, Westmead, NSW 2145 Australia

**Keywords:** Genetic, Bone, Fragility, Osteogenesis, Imperfecta, Nosology, Dyadic

## Abstract

In 2023 following extensive consultation with key stakeholders, the expert Nosology Working Group of the International Skeletal Dysplasia Society (ISDS) published the new Dyadic Nosology for Genetic Disorders of the Skeleton. Some 770 entities were delineated associated with 552 genes. From these entities, over 40 genes resulting in distinct forms of Osteogenesis Imperfecta (OI) and Bone Fragility and/or Familial Osteoporosis were identified. To assist clinicians and lay stake holders and bring the considerable body of knowledge of the matrix biology and genomics to people with OI as well as to clinicians and scientists, a dyadic nosology has been recommended. This combines a genomic co-descriptor with a phenotypic naming based on the widely used Sillence nosology for the OI syndromes and the many other syndromes characterized in part by bone fragility.

This review recapitulates and explains the evolution from the simple Congenita and Tarda subclassification of OI in the 1970 nosology, which was replaced by the Sillence types I–IV nosology which was again replaced in 2009 with 5 clinical groups, type 1 to 5. Qualitative and quantitative defects in type I collagen polypeptides were postulated to account for the genetic heterogeneity in OI for nearly 30 years, when OI type 5, a non-collagen disorder was recognized. Advances in matrix biology and genomics since that time have confirmed a surprising complexity both in transcriptional as well as post-translational mechanisms of collagens as well as in the many mechanisms of calcified tissue homeostasis and integrity.

**Supplementary Information:**

The online version contains supplementary material available at 10.1007/s00223-024-01248-7.

## Introduction

Since 1970, various international committees have guided development of the Nosology of Genetic Disorders of the Skeleton, including osteogenesis imperfecta (OI) and similar disorders characterized by bone fragility [1]. The Nosology subcommittee of the ISDS, composed of international experts in paediatric radiology, clinical genetics, anatomical pathology, endocrinology, matrix biology and clinical genomics, has assumed this role since 1992 [2]. The committee has met every four years to update the Nosology of Genetic Skeletal Disorders. The 11th International revision in 2023 proposed a dyadic classification of OI and the Brittle Bone syndromes [3]. The dyadic nosology comprises a broad phenotypic description based on an expanded Sillence classification, linked to a specific known gene, in which a genomic variant, results in a specific brittle bone phenotype.

The 2023 Nosology distinguished between syndromes of OI (intrinsic bone fragility) and a large group of disorders with bone fragility resulting from heritable types of osteoporosis, singly or as a feature of a multi-trait heritable syndrome e.g. Spondylo-ocular syndrome resulting from genomic variants in *XYLT2*, On-Line Mendelian Inheritance in Man (OMIM*):* 605822 [4]*.*

In this review, the use of ‘‘we/our’’ refers to research by the author, David Sillence and his collaborators.

## The ‘‘Sillence’’ Clinical Classification

### What was the Evidence for the Clinical Classification?

Between 1975 and 1977, a population and genetic study of subjects with OI and familial bone fragility informed by clinical, radiographic, genetic, and in some individuals, histopathologic findings was undertaken in Victoria, Australia [5–7]. The study set out to examine and document the features in as many family members as possible. Families were visited in their home and community settings as well as during hospital outpatient visits. In families where a child was deceased, it was possible to examine the newborn or childhood radiographs and autopsy reports as well as to interview the families. The 180 patients ascertained from the whole population studies fell into four syndromic groups.

The syndromes were not numbered initially, but in 1978 Dr Victor McKusick asked that the syndromes be numbered with Roman Numerals along the lines of the nosology for Ehlers-Danlos syndromes in order to include them in the *Mendelian Inheritance in Man* (MIM) catalogue. The four groups proposed were, Dominantly Inherited OI with Blue Sclerae which became OI type I, Perinatally Lethal OI (OI type II), Progressively Deforming OI (OI type III) and Dominantly Inherited OI with Normal Sclerae (OI type IV) [7, 8]. There was strong evidence for autosomal recessive inheritance in some sibships of OI type II and OI type III. Although further genetic heterogeneity was observed to be likely within OI groups 2 and 3, the nosology has evolved from that point. The observation was made that, although there was significant intrafamilial clinical variability within each of the 4 groups, these were sufficiently distinct from each other from a clinical and genetic perspective. OI type I was distinguished from OI type IV by the presence of distinctly blue sclerae and a high frequency of young adult hearing impairment in the former. The clinical nosology was developed before a biochemical basis for OI was known.

Between 1977 and 1980, an expanded study of the genetic heterogeneity in OI was undertaken through the clinic records of the Orthopedic Hospital Los Angeles (LA) and the Shriners’ Hospital System in USA. A further 250 patients with OI were ascertained. Review of the clinical features, family histories, radiology and pathology in 78 cases confirmed the observation that there were ‘‘at least four types (groups) of OI, but that there was a further confirmation of a possible X-Linked recessive type of OI’’ and a subgroup of OI type IV (now known as type 4) patients who developed hyperplastic callus (Sillence 1980—Report to the Osteogenesis Imperfecta Foundation Research Committee)*.* There was insufficient genetic and phenotypic differentiation in 1979 to confirm that the hyperplastic callus type was a further distinct entity i.e. OI type V (now known as type 5). In 1984, a large series of cases with the Severe Perinatal forms of OI was published [9]. In addition, the studies clarified and extended the clinical experience of individuals and families with OI type III and OI type IV [10, 11].

Dr Jurgen Spranger reported these research studies at the first International Conference on OI in Oxford in 1981 and gave a very clear explanation that the numbers were provisional syndromes, not a severity grading (J Spranger 1981, personal communication). Subsequently he drew attention to the fact that occasionally infants with OI type II with fewer rib fractures did survive longer term and that some families with dominantly inherited OI type 4 included individuals who might have been ascertained as having sporadic OI type III.

The wide phenotypic heterogeneity observed in population studies of children and adults with bone fragility disorders was not new. Knud Stakeman Seedorf had made a similar extensive study of the OI families in Denmark [12]. He personally visited families at home or in their locality throughout Denmark and meticulously recorded clinical findings in affected individuals. In 1949, he reported on the phenotypic, radiographic and family history features in 180 families. The conclusions were similar to those of the Victorian study. Gunnar Smårs reported on 190 individual patients with OI in Sweden in 1961 [13]. His was a very detailed epidemiologic study throughout Sweden with collection of data on clinical features, fracture frequency and skeletal deformity associated with a detailed evaluation of the socioeconomic impact of OI (jointly with Ragnar Berfenstam). This study also reported on a comparative analysis of the musculoskeletal findings in patients with distinctly blue sclerae versus patients with white sclerae. The study did not examine perinatal or childhood deaths from OI.

The classic text, *The Brittle Bone Syndrome* 1983, was the most comprehensive review of the OI literature up to that time [14]. It included an extensive review of the clinical, radiographic and morphologic study findings focussing on orthopaedic and bone and mineral aspects of OI collected on 333 patients. They were ascertained through 3 centres of expertise, the Nuffield Department of Orthopaedic surgery Oxford, Johns Hopkins University Hospital Baltimore and Berck-Plage Hospital France [14] and spanned all age groups. Fracture history, age of onset, types of deformity in long bones and spine and complications of surgery were extensively recorded. The monograph was presented in such a way to support and expand the conclusions of the Victorian study. Biochemical speculation did not go much beyond research findings in type I collagen biochemistry in OI.

Further population and family studies in New South Wales Australia 1980–1986 confirmed our approach to the phenotypic groupings. In reporting our clinical observations of 358 OI affected to an NIH symposium in 1986, we included as probably OI type 3 only those individuals with a history of progressive deformity and normal sclerae where there was sibling recurrence and/or parental consanguinity in 8 families (2.2%) [15]. One hundred and fourteen (31.2%) had familial OI type I and eighty seven (24%) had familial OI type IV. This left a large group of individuals ascertained with a provisional diagnosis of OI with normal sclerae in a group recognized as UNCERTAIN often because of their young age, the uncertainty arising because they may have had OI type IV or OI type III. In retrospect, some families had OI type 5 and other rare brittle bone syndromes (see below). OI phenotyping was always an evolving challenge.

### Why Organize the Nosology in This Way

Following the publication of the clinical and genetic classification in 1977 and its widespread adoption, a large number of predominantly heterozygous pathogenic variants in type I collagen genes *COL1A1/COL1A2* were reported. It was not until 2003 that the first autosomal recessive disorder in *PLOD2* coding for Lysyl hydroxylase 2 was discovered, soon to be followed by multiple loci coding for processes of post-translational modification of type I collagen polypeptides. At the 2009 meeting of the nosology advisory group of the ISDS, a decision was made to group the known OI syndromes into 5 groups [2, 16]. This preserved the primary four types (groups) delineated in the 1970s and added the fifth type of OI, OI with Calcification of Interosseous Membranes, which was confirmed to have a distinct histomorphometric phenotype [17]. The newer disorders and all the known molecular defects which further extend the genetic heterogeneity were encapsulated as subtypes of one of these phenotypic groupings, (Tables 1, 2, 3, 4, 5, 6).

**Table 1:** Osteogenesis Imperfecta Non-deforming with Blue Sclerae (Sillence OI Type 1) Synonym: Van der Hoeve Syndrome

Disorder—Gene	Mode of inheritance	MIM No
OI type 1, *COL1A1*-related	AD	166200 166240
OI type 1, *COL1A2*-related	AD	166200 166240
OI type 1, *COL1A1/COL1A2*-related—high bone mass	AD	
OI type, *PHLDB1*-related	AR	620639

**Table 2:** Osteogenesis Imperfecta Severe Perinatal (Sillence OI Type 2)

Disorder—Gene	OMIM Type name	Mode of inheritance	MIM No
OI type 2, *COL1A1*-related	II	AD	166210
OI type 2, *COL1A2*-related	II	AD AR	166210 259400
OI type 2, *CRTAP*-related	VII	AR	610854
OI type 2, *P3H1*-related	VIII	AR	610915
OI type 2, *PPIB*- related	IX	AR	259440
OI type 2, *TAPT1*-related	OCLSBG**	AR	616897

**OCLSBG = Osteochondrodysplasia, complex lethal, Symoens-Barnes-Gistelinck type

**Table 3:** Osteogenesis Imperfecta Potentially Progressively Deforming (Sillence OI Type 3)

Disorder—Gene	OMIM Type name	Inheritance	MIM No
OI type 3, *COL1A1*-related	III	AD AR	259420
OI type 3, *COL1A2*-related	III	AD AR	259420
OI Type 3, *SERPINF1*-related	VI	AR	613982
OI type 3, *CRTAP*-related	VII	AR	610682
OI type 3, *P3H1*-related	VIII	AR	610915
OI type 3, *PPIB*-related	IX	AR	259440
OI type 3, *SERPINH1*-related	X	AR	613848
OI type 3, *FKBP10*-related	XI	AR	610968
OI type 3, *SP7/OSX*-related	XII	AR	606633
OI Type 3*, BMP1*-related	XIII	AR	614856
OI Type 3, *TMEM38B*-related	XIV	AR	615066
OI Type 3, *WNT1*-related	XV	AR	615220
OI Type 3, *CREB3L1*-related	XVI	AR	616229
OI Type 3, *SPARC*-related	XVII	AR	616507
OI Type 3, *TENT5A*-related	XVIII	AR	617952
OI Type 3, *MBTPS2*-related	XIX	XLR	301014
OI Type3, *MESD*-related	XX	AR	616294
OI Type 3, *KDELR2*-related	XXI	AR	619131
OI Type 3, *CCDC134*-related	XXII	AR	619795
OI Type 3, PLOD2^**^-related		AR	609220
OI Type 3, SEC24D^*^-related^*^		AR	616294

*Mutations in *SEC24D* result in OI with craniosynostosis like features, a Cole-Carpenter syndrome-like presentation

**Mutations in *PLOD2* result in Bruck syndrome type 2 (Osteogenesis Imperfecta with Congenital Joint Contractures

**Table 4:** Common variable OI with normal sclerae (Sillence OI Type 4)

Disorder—Gene	Mode of inheritance	MIM No
OI type 4, *COL1A1*-related	AD	166220
OI type 4, *COL1A2*-related	AD	166220
OI type 4, *CRTAP*-related	AR	610682
OI Type 4, *PPIB*-related	AR	259440
OI Type 4, *SP7*–related	AR	606633
OI Type 4, *PLOD2*-related	AR	609220
OI Type 4, *FKBP10*-related	AR	610968
OI Type 4, *MBTPS2*-related	XLR	301014
OI Type 4, *KIF5B*-related	AD	602809

**Table 5:** Mutations in IFITM5- OI type 5

Disorder—Gene	Mode of Inheritance	MIM of condition	Gene Product
OI type 5, *IFITM5*-related	AD	610967	MALEP* insertion into BRIL**
OI type 6-*IFITM5*-related-pS40L	AD	610967.002	BRIL**- p.Ser40Leu

*MALEP insertion = Methionine-Alanine-Leucine-Glutamic acid-Proline

**BRIL = bone-restricted interferon-induced transmembrane-protein-like

**Table 6:** Familial Osteoporosis and Special Syndromes with Phenotypic features overlapping OI

Disorder	Mode of inheritance	MIM No
X-Linked Osteoporosis, *PLS3*-related	XLR	300910
X-Linked Osteoporosis,-*MBTPS2*-related	XLR	301014
AD Osteoporosis, *WNT1*-related	AD	615220
AD Osteoporosis—*LRP5*-related	AD	166710
AD Osteoporosis—*ARHGAP25*-related	AD	610587
Osteoporosis Pseudoglioma—*LRP5*-related	AR	259770
Spondylo-ocular Dysplasia-*XYLT2*-related	AR	605822
OI with Congenital Joint Contractures Type 1 (Bruck Syndrome)—*FKBP10*-related	AR	259450
OI with Congenital Joint Contractures Type 2 (Bruck Syndrome)—*PLOD2*-related	AR	609220
Familial Osteoporosis with Calvarial Doughnut Lesions—*SGMS2*-related	AR	126550
Osteoporosis with radiolucent lesions of the mandible-*ANO5*-related Gnathodiaphyseal Dysplasia	AD	166260
Osteogenesis Imperfecta with craniosynostosis (Cole-Carpenter)—*P4HB*-related	AD	112240
Osteogenesis Imperfecta with craniosynostosis (Cole-Carpenter Like)—*SEC24D*-related	AR	616294
Singleton-Merten syndrome type 1—*IFIH1*-related	AD	182250
Singleton-Merten syndrome type 2—*DDX58*-related	AD	616298
Short Stature, Bone fragility, DD and Immunodeficiency—NBAS* related	AR	614800*
Snyder-Robinson Syndrome—*SMS*-related	XLR	309583

*Neuroblastoma Amplified Sequence (see SOPH syndrome)

The 2009 ISDS committee retained the Sillence classification ‘‘as the prototypic and universally accepted way to classify OI’’ [16]. To distinguish the Sillence types from the OMIM genomic cataloguing, the ISDS committee recommended that the Sillence OI types be written with Arabic numerals. Namely OI type 1, type 2, type 3 and type 4. Notwithstanding the ISDS Nosology committee’s approach, the need to unequivocally describe the molecular complexity has resulted in the allocation of numerical identifiers in OMIM (characteristic Roman numerals). This is acknowledged in several recent research reviews which correlate genomic with recent advances in cell biology and investigation of bone quality [18, 19].

As OMIM is a gateway to multiple linked genomic, protein and interspecies biochemical databases, the 2023 ISDS Nosology, apart from retaining a clinical correlation with an expanded ‘‘Sillence’’ grouping, has linked the syndromes to their genomic origins, creating a dyadic nosology.

## Phenotyping

Phenotyping and syndrome diagnosis require recognition that some clinical features and histories change over time. Enquiry about fractures must recognize that bone fragility may be manifest prenatally or at any time of life. Furthermore, bone fragility may only be revealed in the spine on X-ray and radiographic studies may appear normal even to the trained eye. Various types of deformity of long bones and spine are typical of OI and familial osteoporosis/or syndromic forms of Bone Fragility disorders. Joint contractures, particularly when present from birth may be a feature of the Bruck syndromes, type 1(resulting from *FKBP10* pathogenic variants) or type 2 (resulting from *PLOD2* variants) [20]. Joint contractures may develop in many other types of OI particularly where there has been immobilization.

### Fracture History and Bone Fragility

The International Society for Clinical Densitometry in its reports in 2013 and 2019 discusses the positive predictability of vertebral fractures (VF) in the suspicion of diagnosis of Osteoporosis/Bone Fragility. In a cohort of children with OI type 1 and predicted *COL1A1* haploinsufficiency, 75% of children were found to have VFs. On the other hand, non-vertebral and long bone fractures are relatively common in children. As a rule, suspicion of OI or a related bone fragility syndrome should arise where there are > 2 long bone fractures by 10 years and > 3 long bone fractures by 19 years of age [21, 22].

### Blue Sclerae

‘‘Early in our original study of Osteogenesis Imperfecta (1975–1977), a most striking observation was made, the most severely deformed patients had sclerae of normal colour’’ [7, 23]*.* This was not a novel observation, as a report from the Nuffield Department of Orthopaedic Surgery in Oxford had reported this observation in 1975 [24]. McKusick 1972 cites Axman (1831) as probably the first to report this observation [25] and there have been multiple scholarly contributions since. In fact in a review of 190 patients (160 with blue sclerae), Smårs had reported a detailed analysis of severity contrasting findings in people with OI with distinctly blue sclerae versus normal sclerae[13]. Patients with distinctly blue sclerae had fewer fractures at birth, less deformity of long bones and spine but a higher frequency of young adult-onset hearing loss than subjects with normal sclerae. Distinctly blue-grey sclerae are best assessed in daylight but not in direct sunlight. It is such a distinctive feature that if there is any doubt then the sclerae are not blue. Adults with OI type 1 have a high frequency of Arcus Cornea [23].

We observed that the important feature of blue sclerae was commonly overlooked or misreported. One of the young patients who had represented Australia at the International Year of Disabled Persons IYDP 1981 had mild-moderate OI and had distinctly white sclerae. When I commented about his normal scleral hue, he informed me that he was a regular examination patient for student/trainee exams. He informed me ‘‘that he had to tell the students to tell the examiner that he had blue sclerae otherwise they would fail their examination’’.

To undertake our study of patients with OI in Victoria, a 4-year-old female with distinctly blue-grey sclerae was taken to visit an ink manufacturer (Collie & Son Pty) who matched the blue-grey hue in the eyes and compounded the scleral colour which is a unique blue tone. The hue was produced from a mixture of blue, red and black basic tones. Dilutions were made with white pigment to make a chart which was used in our research studies [26]. Incidental follow-up of our young volunteer at the age of 34 years confirmed that the scleral hue remained just as blue. For more precision in assessing scleral hue particularly in institutional or research settings, Zack and colleagues reported a standardized approach to recording the depth of scleral blue-grey hue specifying the ambient lighting, the line of sight of the observer and a comparison with blue shades Munsell charts [27].

Scleral thickness is normal in OI type 1 in contrast to some studies which suggest that in OI type 3/4, scleral thickness may be reduced. There are few ultrastructural studies and no immunochemical and biochemical studies of the composition of the scleral matrix in OI type 1. What we do know is that ultrastructural studies of thin sections of the sclerae in autopsy subjects, stained with lead citrate and uranyl acetate show that there is an increased amount of a fine electron dense granular matrix material reminiscent of cartilage polysaccharides interspersed between the collagen lamellae [28]. Scleral biopsies in life may be risky but the existence of mutant zebra fish carrying genomic variants equivalent to OI type 1, i.e. mutants with nonsense, frameshift or splicing sequence variants in *col1a1a*, *col1a1b* or *col1a2*, could assist to resolve the question about the nature of this matrix component which accumulates in OI type 1 [29].

### The Interpretation of Bone Mineral Density Assessments

Plain radiology was the mainstay of interpretation of skeletal morphology and bone quality. Various nomograms for assessing bone quality were developed including radiographic vertebral morphometry by the Genant method [30] and metacarpal morphometry, but not for infants and young children. Dr Caffey noted 50 years ago that it may not be possible to detect osteoporosis in some types of OI and bone fragility syndromes by plain X-ray, particularly in infants. Prior to the birth of children who are known to be at 50% risk of inheriting a bone fragility genomic variant i.e. where a parent is affected, we caution increased care in handling and recommend against the normal hip exam of the newborn and infant (Ortolani testing), until we can demonstrate that the baby has not inherited the genomic variant.

Radiographic (X-ray) bone changes sufficient to diagnose osteoporosis are not apparent in childhood research studies until bone density (BMD), as measured by Dual Energy X-ray Absorptiometry (DXA) in research studies, falls by 2 standard deviations (about 30% reduced bone mineral content). At the time of first fracture, children with those types of OI which normally do not have bone deformity in the first year of life have skeletal X-ray findings which are not recognized as osteoporosis, yet still have bone fragility. This includes bone fragility arising from specific types of gene sequence variants in *COL1A1*,* COL1A2*, *IFITM5* and other genes. This means a child may be symptomatic with bone fragility before the radiologist can conclude from X-ray assessment that there are progressive changes in long bone or spine bone density consistent with Osteoporosis.

**Fig. 1:**
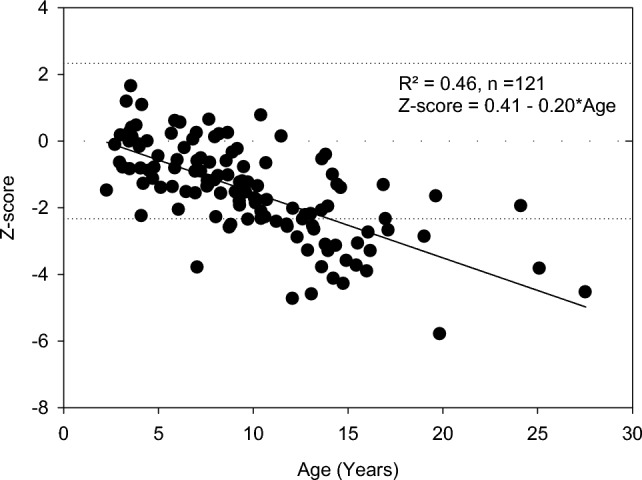
Total Body BMD Z-Scores in OI type 1in children and young adults (*n* = 121)

**Fig. 2:**
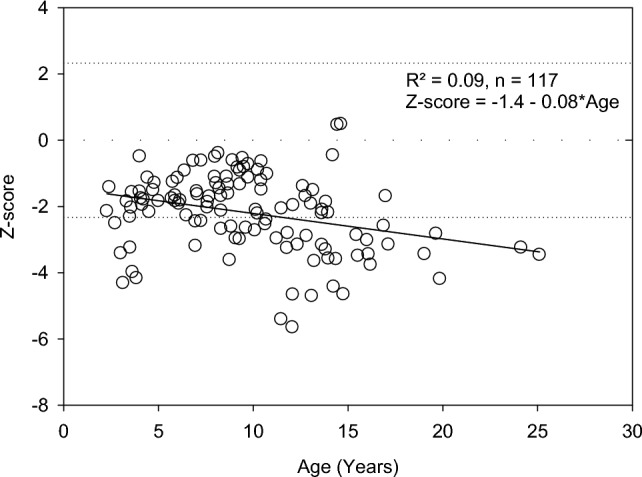
L2-L4 BMD Z-scores in OI type 1 children and young adults (*n* = 117)

At Children’s Hospital Westmead (Westmead, Australia), patients attending the O I Multidisciplinary Clinic had BMD (by DXA) monitored annually from 2 to 3 years of age. To standardize BMD interpretation, commencing in 1992, 266 unaffected children (normal controls) aged between 3 and18 years were recruited and BMD was measured by DEXA (Lunar DPX). The control data were analysed by age, sex, weight and height to determine normal ranges [31]. This enabled the raw DXA data of our OI patients to be converted to Z-scores. Baseline and changes in Z-scores have been used for both bone health assessments and to evaluate the response to various treatment regimens. We also performed DXA assessment on children with various types of OI to assess bone health before they were considered for an interventional therapy (unpublished data). Total and lumbar spine (L2-L4) BMD Z-scores, plotted against age, for children with Osteogenesis Imperfecta type 1 are illustrated in Figs. 1 and 2.

For most children with non-deforming OI aged less than 5 years, Total BMD and half of the L2-L4 BMD values fall into the normal or osteopenic bone density range. Many children younger than 5 years are close to normal or even above. This demonstrates that bone fragility is an inherent characteristic in OI bone. Children with OI may break bones, even when radiographs and DXA bone densitometry measurement show a normal bone density.

**Fig. 3:**
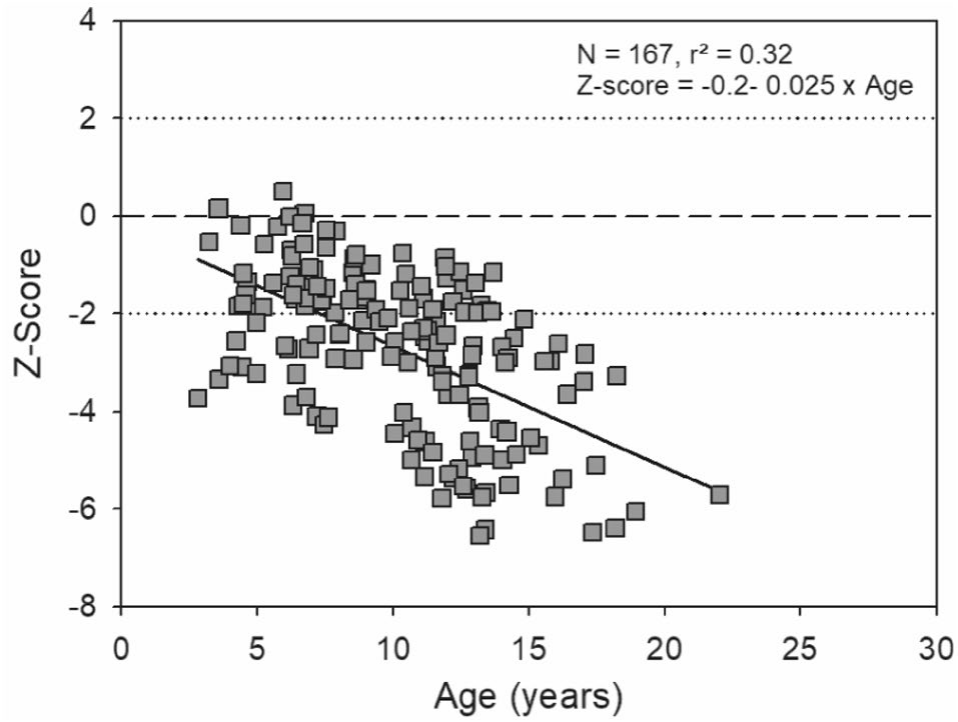
Total Body BMD Z-scores in OI type 3/4 children and young adults (*n* = 121)

**Fig. 4:**
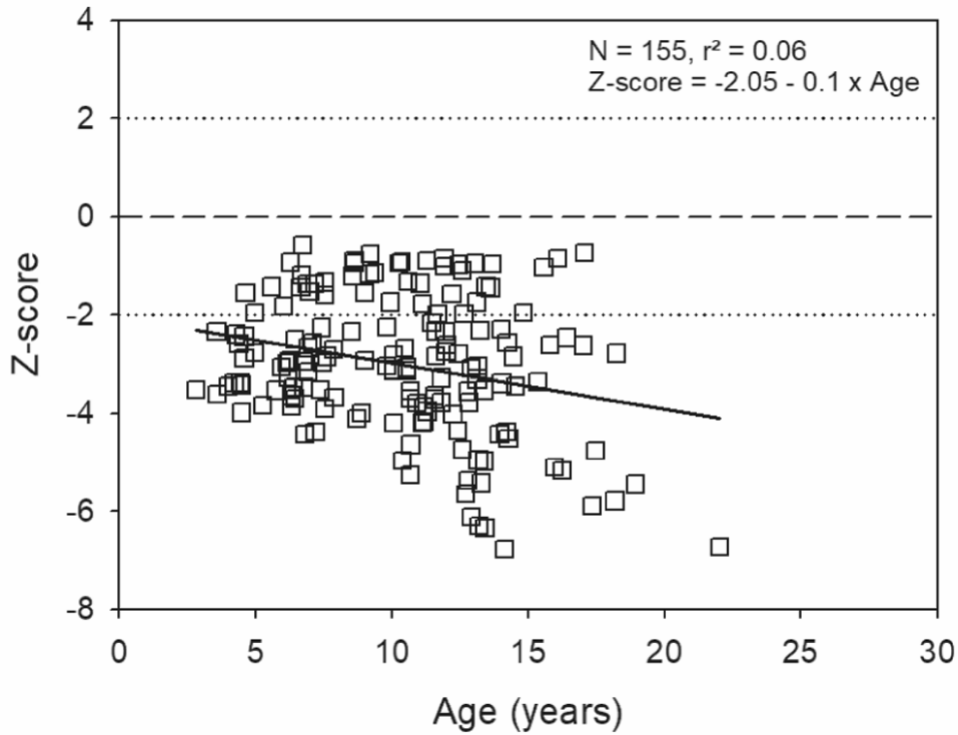
L2-L4 BMD Z-scores in OI type 3/4 children and young adults (*n* = 155)

For other types of OI, osteoporosis may occur earlier in childhood. Figures 3 and 4 show the Total and L2-L4 BMD Z-scores vs age for a cohort of children with OI type 3/4.

As discussed below, this group (Sillence OI type 4) is genetically heterogeneous (Table 4). Patients with missense genomic variants in type I collagen may have a relatively mild bone fragility disorder but there is significant intrafamilial variability resulting from osteoporotic fractures as well as inherent bone fragility fractures.

## Evaluating Severity in OI

Historically the frequency of fractures has been a factor in assessing severity of patients with OI. The most severely affected patients have multiple fractures, often with a history of prenatal and/or perinatal fractures.

To provide a rounded presentation of the recommendations of the Nosology Committee 2013, Fleur van Dijk and David Sillence in 2014 summarized the literature and the approach to clinical diagnosis, nosology and severity assessment [32]. In this research review, they presented a combined textual and visual scale of prenatal and postnatal severity grading, in order to assist health care personnel with evaluation of severity, independently of OI type (Table III in Ref 32). The fracture rate in various groups of OI patients has been partly helpful, although from one individual patient to another there is variability in a family. To create the visual assessment tool, several studies were analysed and employed to provide guidance. These included the 1975–1977 Natural History Study of OI type 1 in Victoria [7] and a natural history study for children with OI type 1 and OI type 4 which concluded that the annualized fracture frequency of children with OI type 1 was 0.49/year.

### Genetic Bases for Bone Fragility

There are many causes of reduced bone mass (osteopenia) and impairment of bone mass sufficient to result in low-impact fractures (osteoporosis). All the disorders discussed in this review have a heritable basis for increased bone fragility. The potential for fractures is life long and the variability is such that a proportion of patients are not diagnosed until adult life, sometimes only when an affected child is born and the parental diagnosis is recognized. In these families, bone fragility, and in many cases osteoporosis, was manifestly heritable and inheritance conformed to Mendelian patterns, Autosomal Dominant (AD) and Recessive (AR) and X-Linked Dominant and Recessive (XL). Prior to 1977, clinicians and researchers proposed that all manifestations of the brittle bone disorders resulted from AD inheritance i.e. heterozygous transmission of various pathogenic variants in the type I collagen genes [25]. AR inheritance was postulated for rare families with sibling occurrence and unaffected consanguineous parents. Formal segregation studies in mainly outbred populations found that for both OI type 2 and OI type 3, there was an approximate 7% chance of sibling recurrence for each group, which was much lower than expected for AR inheritance [33]. Judith Hall in 1988 published a major review of somatic and gonadal cell mosaicism [34], demonstrating sibling recurrence of offspring with serious skeletal phenotypes where the clinically normal parents had preexisting gonadal mosaicism for pathogenic heterozygous genomic variants. Most researchers took the view at that time that AR forms of OI made only a small contribution to sibling recurrence in OI, whereas parental gonadal and sometimes somatic mosaicism for collagen type I variants explained sibling recurrence in OI type 2 and type 3 [35].

Gillian Wallis and Peter Beighton investigating the OI population in Southern Africa discovered that the most prevalent OI phenotype in non-Afrikaans affected people did not segregate with *COL1A1* or *COL1A2* [36]. They proposed that in Southern Africa, the most common moderately severe-to-severe OI phenotype was the result of AR inheritance. Furthermore, they proposed that this autosomal recessive genomic variant was an ancient founder mutation [37]. Vorster and Chetty confirmed that this form of OI plus or minus joint contractures (known as OI type XI or Bruck syndrome type1) resulted from homozygous inheritance of genomic variants in *FKBP10* [37, 38].

Today formal genetic and genomic studies confirm that there are over 24 types of OI and at least 17 familial forms of osteoporosis singly or with extra-skeletal syndromic features resulting in hereditary bone fragility all of which conform to the patterns of Mendelian inheritance and including germinal cell mosaicism. The 2024 ACMG/AMP recommendations for interpreting the significance of genomic analyses require inputs from the findings of clinical and genetic analysis. In many families, family history and pedigree analysis may be a key to interpretation of genomic analysis and the prediction of an evolving phenotype [39].

## Genetic Heterogeneity

The clinician caring for patients with hereditary forms of bone fragility must remember that genomic studies in the last 2 decades have confirmed that the many forms of variability postulated to account for the extensive phenotypic variability in OI observed in the pre-genomic era are now being clarified.

1. OI families show intrafamilial variability. This means that there is V*ariable Expressivity* in severity within families with autosomal dominant types of OI in terms of an effect on stature, number of fractures, joint hypermobility and/or sprains. This variability has been particularly noted in OI type 4 families where there may be a relatively mild disorder segregating in the majority of family members, but where one or two affected family members may appear to have a severe progressively deforming (type 3) phenotype [40]. Potential mechanisms contributing to variable expressivity are discussed in depth in the review by Rivadeneira and Mäkitie in 2016 [41]. Furthermore, there is a commitment to future research and collaboration in the formation of the ‘‘**GE**nomics of **M**usculo**S**keletal traits **T**ranslati**O**nal **NE**twork’’ (GEMSTONE) [42].
2. Different genomic variant mechanisms e.g. nonsense vs missense vs deletional variants in *COL1A1* or in *COL1A2* sequences show considerable *Allelic Heterogeneity*. For example, OI type I has been found to result from premature termination variants, single nucleotide deletions or insertions resulting in a frameshift, or splicing variants in many families. Multi-exon deletional or missense variants may result in an OI type 2, perinatally severe phenotype. Missense variants are found in varying percentages of patients with OI type 3 and dominantly inherited OI type 4 depending on the population studied. In outbred populations without a founder recessive variant, the majority of patients with OI type 3 in the population have been found to have heterozygous variants in type I collagen genes.
3. OI is also characterized by considerable *Locus Heterogeneity* with AD transmitted genomic variants in at least 4 genes, AR genomic variants in 20 genes and X-Linked genomic variants in at least 3 genes.
4. Complex genomic rearrangements such as whole gene, whole exon, multi-exon deletions and also potentially intronic variants may contribute to a bone fragility phenotype.
5. Digenic inheritance has been reported i.e. where expression of genomic variants in 2 separate genes is required, resulting in a bone fragility phenotype more severe or with more complex expressivity than would be accounted for by variants in one of the gene loci operating alone. This is demonstrated for a number of genomic variants which operate via the WNT-signalling pathway (see Table 7 and discussion below).

**Table 7:** Other Syndromes of Connective Tissue and Mineralization which present With Bone Fragility and Simulate Osteogenesis Imperfecta

Group/Name of disorder	Inheritance	OMIM
Metaphyseal Dysplasia (Pyle Type), *SFRP4*—related	AR	265900
Hypophosphatasia (Various forms Infantile, *ALPL*—related	AR	241500
Childhood and Odontohypophosphatasia, *ALPL*–related	AR	241510
Adult Hypophosphatasia, *ALPL*–related	AD	146300
Het *WNT3a*–related with Hom *LRP5*	AR	606359
*Het DKK1–*related with Hom *LRP5*	AR	605189
Het *WNT1*–related with Het *LRP5*	AD	164820
Bone fragility with contractures, arterial rupture, and deafness, *PLOD3*–related	AR	612394
Chronic Recurrent Multifocal Osteomyelitis 3, *IL1R1*–related	AD	259860
Cleidocranial Dysplasia, *RUNX2*–related	AD	119600
Cockayne Syndrome A, *ERCC8*–related	AR	216400
Cockayne Syndrome B, *ERCC6*–related	AR	133540

### A Guide to the Proposed Dyadic Nosology for Clinicians

As a note of caution, potential (intrinsic) phenotypes described here may be substantially modified in future decades, as in many countries today, patients including many adults have medically treated and modified OI. This is most commonly the result of effective medical treatment in childhood, primarily through early treatment with antiresorptive medicines such as Cyclic Intravenous Bisphosphonates (abbreviated Bisphosphonate Modified OI) illustrated in Fig. 1.3 in Sillence and Lamandé [43]. Arundel and Bishop (2024) have reviewed the challenges of the past four decades of medical trials while reminding clinicians that while regulatory authorities focus on fracture frequency, there are outcomes of therapy bearing on quality of life which matter also to patients and their families [44].

Although phenotypic heterogeneity of OI was recognized with the drafting of the first International Nosology in 1970 [1], the early focus on pathogenesis was on biochemical variants in type I collagen polypeptides, pro-alpha 1(I) collagen and pro-alpha 2(I) collagen polypeptides. The first definitive collagen anomaly was only reported in 1975 demonstrating reduced synthesis in the pro-alpha 1(I) collagen chain in type I collagen [45]. Between 1975 and 1992, research focussed on investigations of sequence variants in the type I collagens but the variants discovered in these two loci accounted for only 85% of all the genetic variation in OI cell lines investigated to that time. Interest turned to the genetic variations in the genes for enzymes processing and trafficking type I collagens and were rewarded by the discovery of Lysyl Hydroxylase 2 coded by *PLOD2* as a cause of OI with congenital joint contractures (Bruck syndrome type 2). Between 2000 and 2013, new findings in matrix biochemistry and molecular biology and the application of new genomic investigation techniques such as whole exome sequencing (WES) meant that the number of bone fragility gene loci reported doubled between 1997 and 2013 (7 loci). By 2015, 21 loci plus 3 loci in which genomic variants resulting in familial osteoporosis had been discovered. At the end of 2023, variants had been reported in 24 loci resulting in a form of OI plus 3 genes pending and 17 gene loci in which genomic variants result in familial forms of osteoporosis / or in syndromes with bone fragility, such as the Cole-Carpenter syndrome.

Tables and spreadsheets with a dyadic nosology have been prepared in the format of the 2023 ISDS nosology (Tables 1, 2, 3, 4, 5, 6). In keeping with the recommendations of the ISDS Nosology committee, the descriptive groupings have been retained as a guide, alongside the shorthand numerical descriptions written in Arabic numerals. OMIM numbers for each disorder have been included in the tables.

The ISDS committee considered but rejected the reported alternative to the proposed Dyadic Nosology as confusing [18]. It ascribed the phenotypes of OI types I–IV solely to pathogenic variants in type I collagen genes, *COL1A1* and *COL1A2*. This approach proposed that different classes of genomic variants in COL 1 genes alone (nonsense, splicing, deletion frameshift, missense, etc.) determine the full range of phenotypes from severe perinatal through mildly affected non-deforming types of OI. All the other syndromes, with distinct types characterized by Roman Numerals OI type V—OI type XXIII, were listed separately [18].

### Osteogenesis Imperfecta Non-Deforming OI with Blue Sclerae—Sillence Type 1-*COL1A1-*Related and Sillence Type 1-*COL1A2-*Related

OI type 1 is a common form of familial bone fragility which may present in a variety of ways [46–50]. In addition to bone fragility, it is characterized by the distinctly blue-grey scleral hue (virtually 100% penetrant throughout life), joint hypermobility, easy bruising and onset of primarily conductive hearing impairment in young adult life. In our family studies, one in ten affected had not had a fracture at the time of ascertainment, although we have followed up over time noting that bone fragility continues and whether or not it manifests i.e. the extent of **Penetrance** is reflective of skeletal trauma, immobilization osteoporosis and nutritional deficiencies. Some affected children and adults have been referred with primary joint hypermobility and some experience the Joint Hypermobility Spectrum disorder symptoms of fatigue and fibromyalgia. [51]. While dental involvement in many forms of OI is quite variable, in OI type 1, the teeth are either clinically normal or manifest dentinogenesis imperfecta (DI). OI-related DI is more likely to be found where the pathogenic genomic variant is in *COL1A2*. The matrix architecture resulting in a reported reduced corneal thickness is not known, although there are considerable differences in the molecular organisation of the cornea compared with the sclera [52].

Genotypic Heterogeneity in OI type 1 is summarized in Table 1 and partly reflected in differences in variable expressivity resulting from pathogenic variants in *COL1A1* vs *COL1A2* within the group. The most complete resource and bibliography of pathogenic variants responsible for OI type I phenotypes is presently curated by the Leiden databases and can be accessed at the Osteogenesis Imperfecta database https://lovd.nl/OI-genes.

The founder of the Type I collagen variant database which expanded into the OI databases over several decades, Professor Raymond Dalgleish, has collaborated to publish the most current and comprehensive analysis of the variants in 2022 [18]. The clinician should be aware however that some of the clinical correlations were based on misunderstanding of the Sillence nosology. ‘‘Mild and non-deforming’’ did not necessarily indicate Sillence type 1 because genomic studies of families with OI type 4 with normal sclerae had considerable overlap phenotypically apart from the characteristic distinctly blue-grey sclerae which persists throughout the lifetime of people with OI type 1.

Furthermore, a specific presentation with High Bone Mass and Bone Fragility should be noted, which may have a lower fracture frequency [46–50]. These affected individuals may have distinctive deep blue-grey sclerae. Genomic variants are found in the C-propeptide peptidase, the cleavage site and the non-helical C-propeptide [48].

An additional disorder OMIM OI type XXIII has been added to the Sillence type 1 group [53]. This mild-type bone fragility disorder results from biallelic variants in *PHLDB*, a plekstrin homology-like domain family member involved with phosphorylation of multiple processes likely to alter type I collagen biosynthesis. It is further curious that the evolving phenotype involves regressive metaphyseal and vertebral findings reminiscent of metaphyseal anadysplasia. It is noteworthy that spondylometaphyseal radiographic changes are also observed in people with bone fragility arising from *SGMS2* variants [54].

The majority of affected individuals have sequence variants in the coding for the helical region of the type I collagen polypeptides. Approximately, one third of pathogenic genomic variants affect splicing, while nonsense or frameshift variants affect the remaining two thirds of cases. Some children and adults in our population with OI type 1 have reached puberty without symptomatic osteoporosis and have normal bone density studies. We have demonstrated the efficacy of intermittent oral Risedronate, and for some subjects, cyclic intravenous bisphosphonate in effective reversal of osteoporosis. Bisphosphonate therapy may only be required for a restricted period of time particularly during the rapid growth spurt of puberty.

### Osteogenesis Imperfecta Severe Perinatal OI Sillence Type 2-Related

Perinatal presentation of bone fragility occurs associated with many types of OI but Severe Perinatal OI—Sillence OI type 2 is a distinct subgroup of disorders [9, 33]. The genomic heterogeneity is presented in Table 2. Most infants have numerous prenatal fractures commencing from mid-gestation with a radiographic prototype characterized by continuously beaded ribs and crumpled long bones (accordion or concertina appearance). For the most part, specialists in Bone and Mineral Medicine and Orthopaedic surgeons will never encounter this disorder as few babies with severe Perinatal OI survive the first few weeks of life. Counterintuitively, treatment with subcutaneous morphine for pain relief improves ventilation and appears to improve quality of life for an affected baby.

Genomic variants include deletional and pathological missense variants predominantly in the carboxy-terminal third of the *COL1A1* and *COL1A2* genes as well as pathogenic variants in *CRTAP*, *P3H1* and *PPIB*.

On phenotypic grounds, the group was divided initially into 3 groups, the classic form of Severe Perinatal OI with continuously beaded ribs and perinatal respiratory distress (Group A) compared with a group with fewer rib fractures but concertina femora (Group B). Babies with this severity occasionally survive to adult life. There is an approximately 7% sibling recurrence [33]. Parental gonadal cell mosaicism has been demonstrated to account in part for this recurrence rate [55]. A third group have extremely slender ribs and long bones and likely have autosomal recessive inheritance including OCLSBG (Osteochondrodysplasia, complex lethal, Symoens-Barnes-Gistelinck) syndrome.

Paediatricians are tempted to treat babies with severe perinatal OI with anti-resorptive drug therapies, but this is rarely effective. Furthermore, autopsy studies reveal a high frequency of neural migration defects in the brain [56]. In one treated survivor with the less severe variant in our 40 years experience, the young man had intellectual deficits and Attention-Deficit Disorder.

### Progressively Deforming OI—Sillence OI Type 3

In our 1975–1977 population study, about 15% of affected individuals ascertained with bone fragility had a relatively severe type of OI with a history of fracture prenatally or in the first years. They had repeated fractures and progressive deformity of long bones and spine during childhood. They had pale blue-grey to white sclerae, that is a normal sclerae hue for age. Shortening of stature was progressive with age and virtually all adults developed severe kyphoscoliosis. Clinical, radiographic and family history analysis suggested that this group of patients would be genetically heterogeneous [6, 7, 10, 11].

Many patients in the Victorian studies were the only affected in their family, but families may have more than one affected individual suggesting autosomal dominant inheritance with wide expressivity. Autosomal recessive inheritance characterized by sibling recurrence with unaffected parents has been observed in many families and X-Linked inheritance is now well documented in several families. Prior to 2000, approximately, 80% of patients were found to have genomic variants which resulted in missense mutations in the helical regions of type I collagen polypeptides. Between 1980 and 2000, the rapid discovery of pathogenic genomic variants in *COL1A1* and *COL1A2* obscured the body of discovery which suggested that there were other potential defects arising from genomic variants in fundamental mechanisms of post-translational modification and trafficking of collagen proteins as well as in collagen/matrix interactions. A chance collaboration with Dr Annemarie van der Slot from Ruud Bank’s laboratory led to the discovery in 2003 that one type of OI with Congenital Joint contractures, Bruck syndrome type 2, resulted from pathogenic variants in *PLOD2* coding for Lysyl Hydroxylase 2 [20].

A timely review of the collagens and the status of knowledge about post-translational modifications was published in 2004 suggesting that there might be other forms of OI which resulted from mutations in post-translational modifying enzymes and chaperone processes [57].

It was to the credit of Hans-Peter Bachinger and colleagues at the Shriner’s Hospital in Portland who, in a carefully executed study of type I collagen biochemistry, demonstrated the requirements for prolyl 3-hydroxlation (P3H) of procollagen alpha chains [58]. The complex of prolyl-3 hydroxylase 1 (P3H1) with cartilage-associated protein *(CRTAP*) and cyclophilin B (*CYPB*), also known as Peptidyl protein Isomerase *PPIB*, which has peptidyl-prolyl cis–trans isomerase activity, is essential for prolyl 3-hydroxylation.

This multi-protein complex hydroxylates the proline at codon 986 in procollagen α1(I) and at codon 707 in procollagen α2(I) in the 3’ position [59]. In the α1(I) chain, the 3ʹ-hydroxylation is very specific to α1(I) proline 986. All other hydroxyprolines are hydroxylated in the 4ʹ-position. In the OMIM entries, genomic variants in *CRTAP* are identified as OI type VII [59], *P3H1* variants as OI type VIII [60] and *PPIB* variants as OI type IX [61]. In the Dyadic nosology of OI, variants in each of these 3 genes may manifest in the spectrum of bone fragility extending from a progressively deforming phenotype through to a severe perinatal phenotype.

Approximately, 4% of children with a moderately severe OI phenotype with normal scleral hue and normal teeth have a type of OI characterized by a distinctive pattern on bone histomorphometry with excessive osteoid on the trabecular margins and a ‘‘fish scale-like lamellar’’ appearance on polarised light examination. These patients form a distinct subgroup previously designated OI type VI, which is now known to result from mutations in the gene *SERPINF1* [62]. Although not usually identifiable at birth but with frequent fractures following the first year of life, OI type VI is distinctive for increased unmineralized osteoid. There is also an atypical type of OI type VI, in which a specific heterozygous amino acid substitution p.S40L in the *IFITM5* gene results in a pattern of histomorphometric findings identical those resulting from homozygous *SERPINF1* variants [63].

Autozygosity mapping of a mutant gene in a Turkish family with a moderately severe form of OI identified a mutation in the *FKBP10* gene, a molecular chaperone coding for a FK506-binding protein which has peptidyl-prolyl isomerase activity [64]. Variants in *FKBP10* result in a spectrum of brittle bone phenotypes including OI type 3-*FKBP10*-related, designated in OMIM Osteogenesis Imperfecta type XI, as well as OI with Congenital Contractures type1, also known as Bruck Syndrome type 1[65]. This discovery prompted investigation of similar inbred families resulting in the identification of other chaperones.

Since 2006, at least 38 genes in which genomic variations result in OI or familial osteoporosis have been discovered. These genes have all been identified through a combination of techniques of next generation gene sequencing (NGS), autozygosity mapping and bioinformatics. Sequence variants in two genes *PLS3* coding for Plasmin and *MBTPS1* coding for Membrane-bound transcription factor peptidase, site-1, segregate as X-Linked forms of OI/familial osteoporosis. These subjects are said to have sclerae of normal hue and normal teeth. There are only a few families reported to date, but it is likely that there will be further phenotypic variability. Heterozygous females may have reduced BMD on DXA and have fractures.

Doyard and colleagues studying cases with overlap with Stuve-Wiedemann syndrome with bent bones characterized a further form of OI with multiple vertebral compression fractures and blue sclerae resulting from mutations in the gene *TENT5A* coding for Terminal Nucleotidyltransferase 5A previously known as *FAM46A*, *Family with sequence similarity 46*, *Member A* [66].

Families previously diagnosed with Spondylo-ocular Syndrome with autosomal recessive inheritance, moderately severe-to-severe osteoporosis, and eye and hearing involvement without variants in *LRP5*, were discovered through WES to have mutations in Xylotransferase-2 (*XYLT2*) [67]. This led to the exploration of a wider phenotypic spectrum which demonstrated that genomic variants in *XYLT2* gene result in a variable phenotype dominated by spinal osteoporosis, cataract and hearing loss. The features of the Spondylo-ocular syndrome overlap with those resulting from homozygous variants in *LRP5*, the Osteoporosis-Pseudoglioma syndrome [68].

Today the majority of children in the world with OI type 3 and related phenotypes have been or are being treated with a cyclic intravenous bisphosphonate such as Pamidronate, Neridronate or Zoledronate [44]. Therapy is most effective when combined with a multidisciplinary programme of rehabilitation, physiotherapy, occupational therapy, orthopaedic, respiratory and dental care. The aim of antiresorptive therapies is to mobilize the children and add the considerable skeletal benefits of weight bearing and physical activity to pharmacologic therapies. In the pre-bisphosphonate era, 60% of children died from respiratory failure before their 18th birthday. It is almost miraculous to compare the outcomes now for children in the bisphosphonate treatment era who can be commenced on multidisciplinary care including cyclic intravenous bisphosphonate therapy from the first year of life if necessary (see Fig. 1.3 in Chapter 1, in Sillence and Lamandé) [43]. These children with OI type 3 who are treated from birth may be independently weight bearing by the end of the first 3 years of life [43]. Clinicians and families should therefore welcome the future where potentially progressively deforming genomic variants can be effectively modified, and where the life outcome has considerably reduced bone fragility and other complications.

### Common Variable OI with Normal Sclerae in Adult Life, Sillence OI Type 4

Studies of this group of patients with OI characterized by mild-to-moderate bone fragility with sclerae of normal hue also show genetic heterogeneity (Table 4). A large number of families of affected patients ascertained through the Orthopedic Hospital, Rancho Los Amigos Hospital and the Shriner’s Orthopaedic Hospitals had this phenotype. The bone fragility in most affected was of mild-to-moderate severity, with occasional families with at least one affected segregating a more severe (OI type 3) phenotype. Genomic variations in at least 9 genes may present with this mild-to-moderate phenotype. AD inheritance is found with variants in *COL1A1*, *COL1A2* and* KIF5B*, AR inheritance with variants in *SP7*, *CRTAP*, *PPIB*, *PLOD2 *and* FKBP10* and X-Linked inheritance in patients with pathogenic variants in *MBTPS2.*

Two affected adults and 2 affected foetuses with bone fragility resulting from heterozygous pathogenic variants in *KIF5B*, a gene which codes for the heavy chain of Kinesin-1 were reported in 2023. In addition to bone fragility, both adult patients manifested mixed hearing loss and ptosis. Inactivation of kinesin-1 acting in a dominant negative pattern was demonstrated to impair the molecular transport of mRNAs, proteins and subcellular organelles within cells [69].

Paterson and colleagues 1983 reported 48 patients from 15 families with type 4 OI from the UK. These were all familial cases i.e. with at least two members affected in 2 generations [40]. These autosomal dominant families had a higher frequency of Dentinogenesis Imperfecta but a lower frequency of easy bruising, and young adult-onset conductive hearing loss. In a separate study, we noted that there was a higher frequency of basilar impression in those subjects with OI type 4 and Dentinogenesis Imperfecta [70].

The clinical, radiographic and bone densitometry patterns, however, may overlap with Osteogenesis Imperfecta type 5 and familial forms of osteoporosis, such as heterozygous *LRP5* variants and hemizygous *PLS3* variants. In young children with syndromic disorders with manifesting osteoporosis, Table 6 e.g. Spondylo-ocular syndrome, Bruck syndromes types 1 and 2, Gnathodiaphyseal Dysplasia, Familial Osteoporosis with Calvarial Doughnut lesions and Cole-Carpenter syndromes, bone fragility may present prior to the development in the child of the phenotypic traits which characterize some of these disorders. The family history and investigation in family members may be needed to suspect and confirm a diagnosis in these rare syndromes. For example, families with variants in *FKBP10* may have a history of one or more family members with joint contractures and a diagnosis of Arthrogryposis (without a history of fractures), in addition to a family member with bone fragility with minimum or no joint contractures. Knowledge of an increased prevalence of a particular type of OI in a population and in particular knowledge that there is a founder mutation for an autosomal recessive disorder in that population may guide genomic testing.

## Families Designated OI Type 3/4

As discussed previously, there are families where there is a continuity in the spectrum of severity between those affected with a predominance of affected persons with mild-to-moderate severity but an occasional affected individual presenting with a progressively deforming phenotype [40]. Variability in severity may reflect the impact of postnatal experiences such as severity of fractures, immobilization osteoporosis, previous surgeries as well as constitutional factors. Apart from studies in populations where there is a high frequency of carriers of one of the rare autosomal recessive types of OI, such as FKBP10 in southern Africa, there are insufficient reports of the variability of manifestations in patients with OI type 3 and OI type 4 to draw conclusions about mechanisms of variable expressivity.

### *IFITM5* Related Disorders—Osteogenesis Imperfecta with Calcification in Interosseous Membranes ± Hyperplastic Callus—OI Type 5

While an OMIM number was assigned to this syndrome only in the year 2000, the association of OI with hyperplastic callus following a fracture was first described by Battle and Shattock in 1908 [71]. Over the past 100 years, the reports of the association of OI with hyperplastic callus have been consistent in their description of the phenotypic findings. Fairbank and Baker (1948) reported the following observations in six patients with OI in whom hyperplastic callus occurred [72].

- Formation of intensely calcified local callus was not invariably preceded by a recognisable recent fracture or surgery.
- Excessive ossified callus enveloped the shaft to a varying extent.
- Formation of bony excrescences on the shafts of long bones, particularly on the interosseous borders and without the faintest sign of antecedent fractures.

Bauze and colleagues (1975) reported two families with parent to child transmission of hyperplastic callus, a mother 38 years old and her 6-month-old son and a father 41 years and his 2-year-old daughter [24]. The paper by Bauze and colleagues reports 45 patients with what we would now call a Common Variable type of OI with normal sclerae (OI type 4). Ten Percent (10%) of subjects with moderate-to-severe OI and normal sclerae were found to have this type of OI with linear exosteal ossification. In the histomorphometric study of OI type 4, from Montreal, 7/26 cases (25%) were detected with an abnormal bone histomorphometry. These studies clarified that the clinical diagnosis was made ‘‘over time’’ i.e. the exosteal hyperostosis/calcification of interosseous membranes is a progressive feature. Kozlowski and Bittner had proposed this as a new type of OI in 1981 [73]. A submission was made to the ISDS Nosology in 1996 to include this disorder as a further distinct type of OI. Glorieux and colleagues in 2000 demonstrated that there was a distinctive iliac crest bone histomorphometric pattern with coarse mesh-like lamellation of trabecular bone which added to what had been widely accepted as a separate entity [17].

OI type 5 is inherited as an autosomal dominant disorder and shows variable penetrance and expressivity. The classic variant in *IFITM5*, c.–14 C > G which inserts a new upstream start codon, and results in an N-terminal extension of 5 amino acids (Methionine-Alanine-Leucine-Glutamic acid-Proline = MALEP) into the bone-restricted interferon-induced transmembrane-protein-like (BRIL) molecule [74]. While the disorder is often identified through patients with a moderate-to-severe bone fragility disorder, family members segregating the mutation may have an extremely mild phenotype with no history of fracture. Clinically diagnosable OI type 5 accounts for approximately 5% of individuals with OI seen in the hospital setting. The calcification of the interosseous membranes in the forearms is observed from early in life, which leads to restriction of pronation and supination, and eventual dislocation of the radial heads. Calcification of the interosseous membranes between tibia and fibula is also found in about 20% of patients. The sclerae are white and teeth are normal. Wormian bones are not present. Those affected tend to have higher serum alkaline phosphatase values and have an increased risk of developing hyperplastic callus following a fracture or orthopaedic surgery. Familial recurrence is consistent with autosomal dominant inheritance. Hyperplastic callus is a rare medical emergency occurring in patients with OI type 5. In these situations, a massive callus develops, leading to swelling and pain at the site of the fracture, which can mimic osteosarcoma, but may be distinguished from it on MRIand CT. Its progress can be prevented by the prompt use of indomethacin, an anti-inflammatory prostaglandin inhibitor.

## Genomic Variants in *IFITM5*

There is a further variant in *IFITM5*, the heterozygous p.(S40L) (p.Serine40Leucine) which results in a phenotype which would clinically and histomorphometrically be characterized as a form of OI type 6 [63]. Thus, in some instances, a specific genomic variant may specify a special bone fragility syndrome e.g. the specific type of Osteogenesis Imperfecta type 6, *IFITM5*-related—p.S40L variant.

### Bone Fragility, Osteopenia But Hypermineralization of Bone

In their study of mineralization in growing children with mild Osteogenesis Imperfecta, Roschger and colleagues examined iliac bone by quantitative backscattered electron imaging (BEI) bone histomorphometry [75]. They reported their findings in two groups. Subjects with distinctly blue sclerae with genomic variants resulting in a quantitative defect in gene expression were compared to a group with predominantly normal sclerae who had qualitative defects in type I collagen, predominantly familial *COL1A2* genomic variants. All patients, both those with OI type 1 (distinctly blue sclerae) and those with normal sclerae (OI type 4), had hypermineralization.

Further studies involving not just the Sillence types resulting from pathogenic genomic variants in *COL1A1* and *COL1A2* but also patients with OI type 5 and 6 and many of the OI numerical types characterized by variants in genes coding for enzymes involved in post-translational modifications of type I collagen proteins (such as *SERPINF1*, *CRTAP*, *P3H1*, *SERPINH1*, *FKBP10)*, have been found where the application of BEI to thin sections of biopsied iliac crest is characterized by hypermineralization of bone tissue [19]. This has led to the hypothesis that ‘‘hypermineralization of bone tissue is a cardinal unifying feature of most types of OI’’. This is a major tissue property underlying OI bone brittleness, which refers to susceptibility to fracture by snapping when bent or displaced beyond the yield point [76].

The identification of higher than average mineralization densities than age and site matched normal control specimens (hypermineralization) is also in agreement with studies which had shown abnormal collagen assembly and higher proportion of non-collagenous matrix components when bone biopsies were studied by high-resolution transmission electron microscopy in selected OI patients [77].

However, hypermineralization is not found in biopsied bone from patients with variants in *SGMS2* or A*RHGAP25* (O Mäkitie, personal communication 2023). Given that these patients, particularly in early childhood, may have no extra-skeletal clinical findings of OI, the clinician may not easily clinically distinguish them from affected individuals with heterozygous pathogenic variants in *COL1A1* and *COL1A2*.

### Familial Osteoporosis and Bone Fragility in Rare Genetic Syndromes

Patients with familial forms of osteoporosis (OP) may present with bone fragility with normal sclerae and this suggests a diagnosis of Osteogenesis Imperfecta type 4, yet the osteoporosis may have a very different pathogenesis. Table 6 in the 2023 nosology includes X-Linked OP, *PLS3*-related and X-L OP, *MBTPS2*-related (also known as OMIM OI type XIX, *MBTPS2*-related as well as three autosomal dominant forms of Osteoporosis, OP, *WNT1*-related, OP, *LRP5*—heterozygous-related and OP, *ARHGAP25*-related. The autosomal dominant disorder resulting from a heterozygous missense variant in the gene *ARHGAP25* which codes for Ras homologous guanosine triphosphatase (RHOGTPase) -activating protein 25 canonical transcript isoform 4 does not yet have an OMIM assignment [78]. Affected family members have bone fragility from mid-childhood extending into adult life, normal sclerae, normal hearing and normal BMD. Bone fragility is associated with abnormal bone histomorphometry revealing severely reduced cancellous bone volume and parameters of reduced bone turnover.

Bone fragility occurs in a large number of other rare syndromes as well as being associated with a number of Inborn Errors of Metabolism (Table 6). These include the syndromes with eye involvement including Osteoporosis Pseudoglioma Craniosynostosis (Cole-Carpenter and Cole-Carpenter Like), with congenital joint contractures (Bruck syndromes), Calvarial lucencies, Jaw lesions (Gnathodiaphyseal), X-Linked Intellectual Disability (Snyder-Robinson), and Immunodeficiency plus or minus hepatic failure (*NBAS*-related). A diagnostic investigation or radiographic handle is not always apparent in young children and minimum-trauma fractures may occur without warning. This is also very true of the large number of disorders which may manifest with transient neonatal hyperparathyroidism (TNHPT) Supplementary Table 9.

The extraordinary allelic heterogeneity and pleomorphic manifestations of genomic variants in Low Density-Related Protein-5 (*LRP5)* and *LRP6* need special mention. Initially research focussed on the autosomal recessive syndrome of Osteoporosis Pseudoglioma plus or minus Mental retardation in which genomic variants in *LRP5* were well characterized. However, with the application of NGS investigations, a wide diversity of pleomorphic complications in addition to both Familial AD and AR osteoporosis have been documented [79–81]. These include Familial Type 2 Diabetes, and Familial Exudative Vitreoretinopathy (FEVR) [82, 83].

The 2023 Nosology also includes a number of rare syndromes in other ISDS pathogenetic groupings which may result in those affected presenting for diagnosis because of bone fragility (Table 7). These include all forms of Hypophosphatasia, Cleidocranial Dysplasia-*RUNX2*-related and Cockayne syndromes A and B. There are also recent reports of digenic inheritance of osteoporosis resulting in early-onset osteoporosis.

## Digenic Inheritance of Osteoporosis

Homozygous pathogenic sequence variants in *WNT1* may result in a severe, possibly life terminating brittle bone disorder, presenting in infancy with cerebellar malformation and the syndrome is allocated OMIM OI type XII. The heterozygous *WNT1* variants result in Familial Osteoporosis except that, as further patients are reported with pathologic genomic variant in *LRP5* plus a synergistic variant in a second locus such as W*NT1*, *DKK1*, *WNT3A*, *SFRP4*, the pathogenesis of the familial osteoporosis spectrum of disorders is widened [82].

Caetano da Silva and colleagues (2021) have reported the influence of variants in *DKK1* or *WNT3A* on phenotypic expression of biallelic pathogenic variants in *LRP5* [82] (Table 7). This is important as ancillary genomic testing for variants in synergistic WNT-signalling loci is not routinely curated when variants are found in *LRP5* [82]. The authors recommend that these loci be included in genomic panels for investigating Early Onset/Familial Osteoporosis and Bone Fragility. The Quantitative Trait Locus (QTL) Association literature has many examples of the additive effects of bone—influencing phenotypic expression of variants and there is no reason to not expect to observe these synergistic interactions in subjects with Osteogenesis Imperfecta and related disorders [41].

As a special note, digenic inheritance of genomic variants in WNT-signalling pathways may manifest as if they are operating as a monogenic trait.as a result of co-segregation from a parent to child.

An updated Premature Ageing group (Supplementary Table 8) has been added. Over 20 premature ageing disorders associated with bone fragility and manifest from the perinatal period through to young adult life have been delineated and their pathogenesis elucidated. Cerebroretinal microangiopathy with calcifications and cysts (CRMCC) resulting from genomic variants in *CTC1* is included as this disorder, like Hutchinson-Gilford Progeria, is strongly associated in affected individuals with osteopenia/osteoporosis and recurrent stress fractures throughout life [84].

Supplementary Table 9 summarizes the genomic basis for Transient Infant Bone Fragility resulting from Transient Neonatal Hyperparathyroidism (TNHP) which has been included because of its significant overlap with early-onset transient bone fragility in babies. It has profound importance for adults where fathers with AD Hypocalciuric Hypercalcaemia may have affected offspring. This group of disorders, (group 27 in the 2023 Nosology), which has been documented in the paediatric and radiologic literature for at least the last 50 years, has been found to result from genomic variants with novel pathogenetic mechanisms in at least 10 genes. In addition, transient infant bone fragility resulting from pathogenic variants in *TRPV6* (Transient Receptor Potential cation channel, subfamily V, member 6), MIM 606680. The underlying disorders result in a wide spectrum of severity of bone changes with bowing, angulation deformity, fractures and periosteal cloaking/mimicking subperiosteal calcification associated with chemical hyperparathyroidism. In most cases, the Neonatal Hyperparathyroidism is transient and resolves spontaneously in the 2nd to 3rd year of life.

### Heterozygous Expression of X-Linked Genomic Variants

Female patients with expression of heterozygous genomic variants in X-Linked *PLS3*, *MBTPS2* and *SMS* may have symptomatic osteoporosis [85]. This could be manifest as childhood onset osteoporosis expressed not only in young males but also females, ‘‘idiopathic’’ osteoporosis, exaggerated pregnancy-related osteoporosis or premenopausal osteoporosis. This is a whole field in itself, and the question is, ‘‘what is the nosologic description of these syndromes of osteoporosis in young adult women with heterozygous variants in *PLS3* or *MBTPS2* or *SMS*’’ in their reproductive years?’’. Some women have pregnancy-related bone loss/reduction in Total Bone Mineral content (TBMC) as the foetus is selectively dependent for building its skeleton by direct maternal resorption and calcium transfer. This mineral transfer occurs in the last 13 weeks of pregnancy and is strongly regulated with *TRPV6* playing a major role [86]. It is significant for heterozygous carriers and women with Osteogenesis Imperfecta of any type [86].

It is important to remember that because of occasional instances of skewed X-inactivation and other genetic mechanisms, even female subjects with heterozygous X-Linked genomic variants e.g. *PLS3* variants may have childhood onset osteoporosis. Mäkitie and Zillikens 2022 observed ‘‘Kampe et al. also described a young girl who presented with recurrent peripheral fractures, extremely low BMD (lumbar spine BMD Z-score − 6.6 at 6 years) and a heterozygous de novo PLS3 variant. This indicates that PLS3 variants should be considered especially in males but even in females with early-onset osteoporosis’’ [79].

## Conclusion

Given the considerable growth of knowledge and the development of further genomic techniques, the next decade of communicating with families with brittle bone disorders and their caring Health Professionals will be challenging. The Clinical Genetics Variant Curation Expert Panel working with the American College of Medical Genetics and Genomics/ American College of Pathologists (ACMG/AMP/CAP/ClinGen) is already working to develop future guidelines for genomic variant interpretation.

## Supplementary Information

Below is the link to the electronic supplementary material.

Supplementary file1 (DOCX 16 kb)
