# Supplementary material for: A Dyadic Nosology for Osteogenesis Imperfecta and Bone Fragility Syndromes 2024
Source: Calcif Tissue Int. 2024 Jun 28;115(6):873–90. doi: 10.1007/s00223-024-01248-7 (PMC11607092; doi:10.1007/s00223-024-01248-7)
Supplement: Supplementary file 1 — Supplementary file1 (DOCX 16 kb) [file 223_2024_1248_MOESM1_ESM.docx]

Supplementary Table 8 Premature Ageing Group

| Disorder | Mode of Inheritance | MIM No. |
| --- | --- | --- |
| Progeria (Hutchinson-Gilford), *LMNA* - related | AD | 176670 |
| Mandibulo-Acral Dysplasia A, *LMNA* - related | AR | 248370 |
| Mandibulo-Acral Dysplasia B, *ZMPSTE24* - related | AR | 608612 |
| Wrinkly skin syndrome, *ATP6VOA2* - related | AR | 278250 |
| Geroderma osteodysplasticum, *GORAB* - related | AR | 231070 |
| Cutis Laxa with Progeroid Features, *PYCR1* - related | AR | 612940 |
| Wiedemann-Rautenstrauch, *POLR3A* - related | AR | 264090 |
| Wiedemann-Rautenstrauch, *COL1A1* - p.(Gly22Arg) related | AD |  |
| Werner Syndrome, *RECQL2* - related | AR | 277700 |
| Metageria (Acrogeria, Gottron type) | AR | 201200 |
| Cerebroretinal microangiopathy with calcifications and cysts (CRMCC), *CTC1* - related | AR | 613129 |

Supplementary Table 9 Bone Fragility Disorders mediated by Disturbance in Maternal-Fetal Calcium Homeostasis. (Resulting in Transient or Severe Neonatal Hyperparathyroidism)

| Group / Name of Disorder | Inheritance | MIM No. |
| --- | --- | --- |
| Transient NHPT Secondary Neonatal Hyperparathyroidism, *SLC12A1* related | AR | 600839 |
| Transient NHPT Secondary to Mucolipidosis II, *GNPTAB* - related | AR | 252500 |
| Transient NHPT Secondary to Galactosialidosis, *CTSA* - related | AR | 256540 |
| Transient NHPT Secondary to Sialidosis type 2 – *NEU1* - related | AR | 256550 |
| Transient NHPT Secondary to Maternal Pseudohypoparathyroidism - *GNAS* - related | AR | 103580 |
| Transient NHPT Secondary to defective Maternal-fetal transfer of calcium – *TRPV6* - related | AR | 618188 |
| Neonatal severe hyperparathyroidism, severe form - (Homozygous inactivating *CASR* variants - related | AR | 239200 |
| Neonatal hyperparathyroidism, severe form - CASR-associated G protein alpha 11 - related *GNA11* | AR | 145981 |
| Neonatal hyperparathyroidism, severe form, (Adaptor Related protein complex 2) *AP2S1* - related | AR | 600740 |
| Familial hypocalciuric hypercalcemia with transient neonatal hyperparathyroidism – *CASR* - related (paternal transmission) | AD | 145980 |

TNHPT = Transient Neonatal Hyperparathyroidism

NSHPT= Neonatal Severe Hyperparathyroidism
